# Integrated Moist‐Thermoelectric Generator for Efficient Waste Steam Energy Utilization

**DOI:** 10.1002/advs.202206071

**Published:** 2023-05-28

**Authors:** Mingchen Yang, Yin Hu, Sijie Zheng, Ziyang Liu, Weizheng Li, Feng Yan

**Affiliations:** ^1^ Jiangsu Engineering Laboratory of Novel Functional Polymeric Materials Jiangsu Key Laboratory of Advanced Negative Carbon Technologies College of Chemistry Suzhou Key Laboratory of Soft Material and New Energy College of Chemistry, Chemical Engineering and Materials Science Soochow University Suzhou 215123 China

**Keywords:** ion migration, moist‐thermoelectric generator, polyelectrolyte membrane, waste steam, wearable devices

## Abstract

Industrial waste steam is one of the major sources of global energy losses. Therefore, the collection and conversion of waste steam energy into electricity have aroused great interest. Here, a “two‐in‐one” strategy is reported that combines thermoelectric and moist‐electric generation mechanisms for a highly efficient flexible moist‐thermoelectric generator (MTEG). The spontaneous adsorption of water molecules and heat in the polyelectrolyte membrane induces the fast dissociation and diffusion of Na^+^ and H^+^, resulting in the high electricity generation. Thus, the assembled flexible MTEG generates power with a high open‐circuit voltage (*V*_oc_) of 1.81 V (effective area = 1cm^2^) and a power density of up to 4.75±0.4 µW cm^−2^. With efficient integration, a 12‐unit MTEG can produce a *V*_oc_ of 15.97 V, which is superior to most known TEGs and MEGs. The integrated and flexible MTEGs reported herein provide new insights for harvesting energy from industrial waste steam.

## Graphical abstract

The highly‐efficient flexible moist‐thermoelectric generator (MTEG) provides new insights for harvesting energy from industrial waste steam. The polyelectrolyte membrane which used the “two‐in‐one” strategy in MTEG spontaneous adsorbs water molecules and heat, which induces the fast dissociation and diffusion of Na^+^ and H^+^, resulting in the high electricity generation.

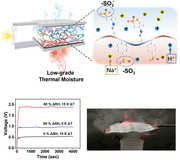

## 1 Introduction

**Figure 1:**
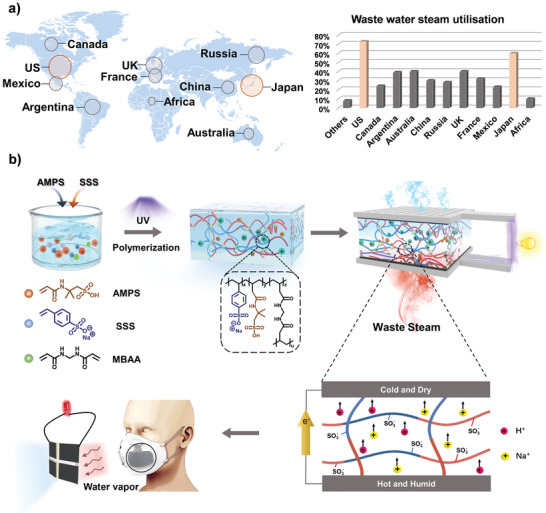
Design and synthesis of the moist‐thermoelectric generator. a) Global waste steam utilization in 2019. Data source: IEA's annual World Balance Sankey Diagram (https://www.iea.org/sankey/#?c). b) Polyelectrolyte membrane was prepared by the copolymerization of AMPS and SSS under UV light. The polyelectrolyte membrane was further encapsulated between carbon cloths with carbon nanotube coating and assembled into the MTEG. The device was capable of generating electricity because of the dissociation and diffusion of Na^+^ and H^+^ under the hygrothermal synergistic system. Hence, it has great application potential in real‐life scenarios, such as masks and hot steam vents.

Fast‐growing industries intensively consume high temperature water steam. However, the effective utilization of water steam is generally low, and over 60% of the energy is dissipated as waste steam (heat) (**Figure** **1**a). For example, the waste steam (heat) from industry is estimated to be nearly 113000 GWh per year in UK, in 2018.^[^ ^1^, ^2^, ^3^ ^]^ While the re‐use of waste steam is usually performed as a heat source for the production process through conventional conversion methods.^[^ ^1^, ^4^, ^5^, ^6^, ^7^, ^8^ ^]^ Therefore, industrial waste steam might be used to generate electrical energy via thermoelectric and moist‐electric conversions.

The route for moisture or thermal energy conversion has been reported in various ionic polymer systems, and the movement of ions under moisture gradient has been identified as the dominant driving force for the conversion of moisture energy in polymer moist‐electric generators (PMEGs). Another kind of high‐performance MEGs is composed of 2D nanomaterials, in particular hydrophilic graphene oxide (GO) owing to the tailorability and low internal resistance. MEGs, as a green energy conversion device, can be applied in the daily field as sensors and power supplies.^[^ ^9^, ^10^ ^]^ Qu et al. reported the efficient MEGs consisting of various polymers with electrolyte groups.^[^ ^11^, ^12^, ^13^, ^14^, ^15^, ^16^, ^17^, ^18^, ^19^, ^20^ ^]^ The spontaneously moist‐electric polymer membrane generator was able to deliver a *V*_oc_ of 0.6 V when poly(4‐styrene sulfonic acid) and poly(vinyl alcohol) were used as the polymer membrane.^[^ ^21^ ^]^ Moreover, a heterogeneous moisture‐enabled electric generator (HMEG) assembled with a bilayer of polyelectrolyte membranes produced a high voltage of approximately 0.95 V, proving that polymers with proton conduction can be effectively applied in the conversion of moisture energy in MEGs.^[^ ^22^ ^]^ However, long‐term moisture supplements cause the elimination of moisture gradients in polyelectrolyte membranes, which results in a low energy conversion efficiency and short duration of output, thus limiting the practical applications of PMEGs.

On the other hand, thermoelectric generators (TEGs) based on the Soret effect, generate electricity through ionic thermodiffusions under temperature gradients.^[^ ^23^, ^24^, ^25^, ^26^, ^27^, ^28^, ^29^ ^]^ Thermoelectric devices, including inorganic‐based, organic‐based, and hybrid‐based thermoelectric power generators and refrigerators, target various applications. Current self‐cooling design can flexibly switch the Seebeck and Peltier effects of thermoelectric devices to achieve the energy harvest and thermal management.^[^ ^30^ ^]^ Poly(ionic liquids) is a good conductor of ions.^[^ ^31^, ^32^, ^33^, ^34^, ^35^ ^]^ Cheng et al. reported that an ionic gel made of ionic liquids and poly(vinylidene fluoride‐co‐hexafluoropropylene) (PVDF‐HFP) exhibited a vast ionic thermovoltage of about 26.1 mV K^−1^.^[^ ^36^ ^]^ Lei et al. designed a double chemically‐crosslinked network gel which exhibited high stretchability. The assembled thermoelectric cell also exhibited an output power density of 0.61 mW m^−1^ K^−2^, which is acceptable considering the limited conductivity and ionic thermovoltage of ionic gels.^[^ ^37^ ^]^ Aqueous thermocells using hydrogel electrolytes containing thermogalvanic ions exhibit high thermal‐electric conversion efficiencies in addition to being eco‐friendly.^[^ ^38^, ^39^, ^40^, ^41^ ^]^ However, water evaporation limits their operating temperature ranges and output stability.^[^ ^42^ ^]^ To solve the existing challenges of humidity saturation for MEGs and water evaporation for TEGs, waste steam energy conversion can be achieved by a moist‐thermoelectric generator which combines the advantages of both thermoelectric and moist‐electric generators.

Herein, we report a “two‐in‐one” strategy that combines thermal‐electric and moist‐electric generation mechanisms to achieve a high *V*_oc_ and large output power density. A polyelectrolyte membrane consisting of a crosslinked copolymer of poly(2‐acrylamide‐2‐methylpropane sulfonic acid) and poly(sodium styrene sulfonate) (PAMPS/PSSS) were prepared, and this membrane with migratable H^+^ and Na^+^ generated electricity under the influence of both temperature and humidity. The efficient proton transport provided fast‐responding electricity. After the humidity saturation of the polyelectrolyte membrane, thermal moisture compensated for moisture evaporation which permitted continuous electricity generation by the thermally mobile Na^+^. Thus, converting low‐grade thermal moisture into continuous electrical energy. The flexible moist‐thermoelectric generator (MTEG) generated power in the hygrothermal synergistic system with a *V*_oc_ of 1.81 V cm^−2^ at 80% humidity and 15 K temperature difference, and achieved a power output of 4.75 ± 0.4 µW cm^−2^ and a high ZT_i_ value of 14.07 ± 1.93 (90% RH, 40 °C). The 12 units MTEGs can produce a *V*_oc_ of 15.97 V. Furthermore, the integrated METGs (sum area = 3 cm^2^) were placed on a mask, and the mask activated a red LED bulb from the energy produced by the hot moisture of human breath. The flexible and wearable MTEG reported herein shows promising prospects for environmental low‐grade thermal moisture energy conversion such as human breath and other real‐life conditions.

## 2 Results and Discussion

### 2.1 Preparation of MTEGs

**Figure 2:**
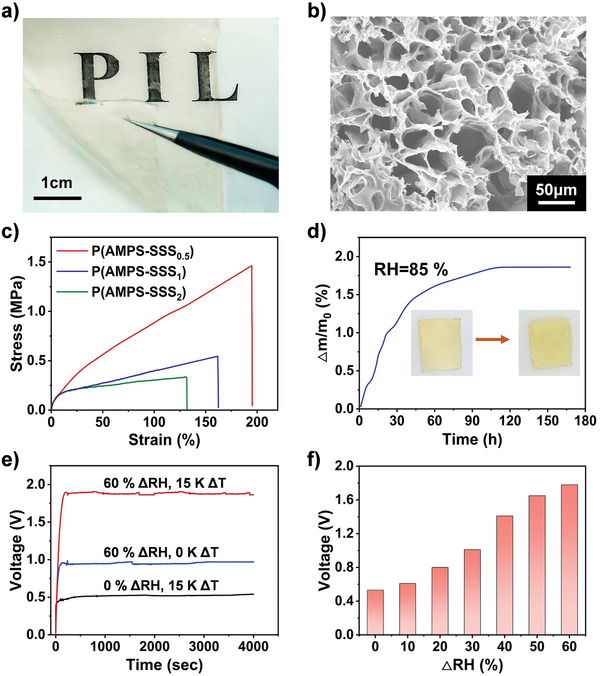
Characterization of the physical properties of the polyelectrolyte membranes and electricity generation ability of MTEGs under different envrionments. a) Photograph of the moist‐thermoelectric polyelectrolyte membrane. b) Scanning electron microscope (SEM) image of the membrane with microporosity. Water vapour percolated through the pores after working for several hours. c) Stress–strain curves of the P(AMPS‐SSS_x_). d) P(AMPS‐SSS_0.5_) water absorbency at 85% relative humidity for 168 h and the inset photographs shows the shape of the membrane before and after the test. e) *V*_ocS_ of the MTEG tested under different environmental conditions. f) Voltages of the MTEG were tested under various humidity differences between two sides of the MTEG.

The polyelectrolyte membranes were copolymerized by 2‐acrylamide‐2‐methylpropane sulfonic acid (AMPS) and sodium styrene sulfonate (SSS). The solution containing AMPS and SSS was polymerized under UV light. The polyelectrolyte film was assembled from carbon cloth and hydrophilic tape to form MTEG which can be used in flexible wearable devices and steam vents (Figure 1b). Attenuated total reflection Fourier‐ transform infrared spectroscopy (ATR‐FTIR) test was performed to investigate the structure characteristics of the polyelectrolyte membranes. The FTIR band at 3300 cm^−1^ is assigned to the amide bonds in AMPS, and the other bands at 1200 and 1100 cm^−1^ are assigned to the ‐SO_3_^−^ in SSS (Figure S1, Supporting Information). The polyelectrolyte membranes were flexible and free‐standing (**Figure** **2**a). As shown in Figure 2b, the membranes were crosslinked with micropores, which are channels for water molecule transport. The different molar ratios of the SSS and AMPS gave the membrane P(AMPS‐SSS_x_) (x = 0.5, 1, 2, respectively) its changeable flexibility. Tensile measurements were used to examine the mechanical properties of the P(AMPS‐SSS_x_) membranes. As shown in Figure 2c, as the molar ratios of SSS increased, the tensile stress and tensile strain increased simultaneously. The intrinsic mechanism is that as the content of SSS increased, the forces of the ionic bonding and the *π*–*π* interactions of the benzene rings between the polymer chains increase. Thus, the obtained P(AMPS‐SSS_0.5_) membrane could be stretched and twisted repeatedly (Figures S2–S4, Supporting Information), and the tensile stress and strain were tested to be ≈1.5 MPa and 200%, respectively. Such a good mechanical property of the P(AMPS‐SSS_0.5_) membrane facilitates the installation of flexible devices. In contrast, both the P(AMPS‐SSS_1_) and P(AMPS‐SSS_2_) membranes were too brittle to maintain stable performance for the MTEGs. Moreover, the mass change (Δ*m*/*m*_0_ × 100%) of the P(AMPS‐SSS_0.5_) membrane by water adsorption from atmospheric moisture (85% RH, 25 °C) was investigated. The mass increased by about 1.8% after 168 h (Figure 2d). The membrane was also virtually unchanged in appearance. All the obtained results coherently reconfirmed the reasonability of conducting wet thermal power generation performance tests on P(AMPS‐SSS_0.5_).

### 2.2 Principle of Power Generation

The flexible MTEGs were assembled as a laminate structure as shown: carbon cloth |P(AMPS‐SSS_0.5_)| carbon cloth (20×20 × 2 mm^3^). A water‐permeable adhesive tape was used as the packaging substrate (Figure S5, Supporting Information). The hydrophilic carbon cloth glued to adhesive tape served as the electrode. The power generation performance of the MTEG in different environments shows that the material is much more efficient in humid‐heat environments than in purely humid or hot environments. The *V*_oc_ of the MTEG reached 1.81 V at a temperature difference of 15 K and humidity differences of ΔRH = 60%, while the system was 0.53 and 0.98 V for the single condition of heat and moisture, respectively (Figure 2e). The temperature difference produced in the system caused the rapid evaporation of water vapor and further enhanced the difference in humidity between the top and bottom. The larger humidity difference increased the output voltage of the MTEG (Figure 2f).

The thermal diffusion of ions under a temperature gradient is also known as the Soret effect.^[^ ^43^ ^]^ For hydrogels, the Soret effect refers to the thermal diffusion phenomenon of the condensed phase. The contributions of the movable cations and anions to the thermal voltage in ion‐driven thermoelectric materials (i‐TE) are similar to the multiband transport in electron‐driven thermoelectric materials (e‐TE). In symmetric ionic electrolytes, temperature gradients drive cations and anions migrations from the hot side to the cold side, resulting in net charge accumulation and internal electric field generation voltages. Based on Onsager transport theory,^[^ ^24^ ^]^ we derived the total ionic thermovoltage of symmetric electrolytes based on Equation (1):

(1) $$S_{i}=\;-\frac{D_{+}{\hat{S}}_{+}\;-D_{-}{\hat{S}}_{-}}{e\left(D_{+}+D_{-}\right)}$$

Here, *e* denotes the meta‐charge, *D*_±_ and ${\hat{S}}_{\pm }$ denote the mass diffusion coefficient and the transfer Eastman entropy of cation or anion, respectively. Therefore, in this P(AMPS‐SSS_0.5_) polyanionic network, the positive ions can migrate with temperature without the negative ions attenuating their thermal diffusion effect. Thus, the ionic interactions induced by a negatively charged network can produce a large transfer Eastman entropy. Meanwhile, a small proportion of the Na^+^ tends to “condense” along the negatively charged polymer chains. This anti‐balance condense was proposed by Manning.^[^ ^44^ ^]^ The movement of water molecules also made the Na^+^ in SSS to immigrate and the H^+^ to dissociate from AMPS, which is the main component of the moist‐electric generation. According to the literature,^[^ ^22^ ^]^ the change in this potential energy corresponds to moving from gaseous water to adsorbed water within the membrane. Therefore, low‐grade thermal moisture was the primary energy source for electricity generation in the MTEG, and this provided the energy for ions dissociation and migration.

In addition, the electricity generation by the MTEG can be quantitatively supported by modeling with coupled Nernst–Planck, and Poisson equations^[^ ^22^ ^]^ and the Onsager transport theory. The Nernst‐Planck equation (Equation (2)) is shown below.

(2) $$j_{i}=\;-D_{i}\left(\nabla c_{i}+\frac{z_{i}Fc_{i}}{RT}\nabla \varphi \right)$$

Where *φ*, *F*, *z*, *c*, *D*, *j*, *R*, and *T* represent the electrical potential, Faraday constant, valence of the ionic species, ion concentration, diffusion coefficient, ionic flux, ideal gas constant, and temperature, respectively. The voltage is related to both the ion concentration and the ion diffusion coefficient, resulting in the non‐linear increase.

According to the experimental results, the output voltage for the hygrothermal synergistic system was greater than the sum of the voltages for the other two separate cases. Both Equations (1) and (2) revealed that the diffusion coefficient of the ions is a crucial factor in the electrical potential of moist‐electricity and thermoelectricity. The mobility of the ions involves the movement of an ion from one site to another through the defects in the crystal lattice of a solid or aqueous solution, and it is the microcosmic expression of the ion's conductivity. Therefore, the mobility of H^+^ and Na^+^ showed an apparent difference indicated by the different electric conductivities. The ionic conductivity of the membrane increased with increasing humidity. In summary, the simultaneous transport of H^+^ and Na^+^ under the synergistic conditions of humidity and heat, increased the ionic conductivity, which further promoted the condensation of ions at the cold side within the system to achieve the “1+1>2” effect.

### 2.3 Properties of Ionic Thermoelectricity

It is known that the efficiency of a thermoelectric cell is reflected by its *ZT_i_* value, which is determined by the ionic thermovoltage (*S_i_*), the ionic conductivity (*σ*_*i*_), and the thermal conductivity (*κ*) (Equation (3)).^[^ ^45^, ^46^, ^47^ ^]^ The measurements of the *S_i_*, *σ*_*i*_, *κ*, power factor (*PF*), and *ZT_i_* were carried out under varying relative humidities to investigate the effect on the P(AMPS‐SSS_0.5_).

(3) $$Z\;T_{i}=\frac{S_{i}^{2}\sigma_{i}T}{\kappa }$$

The ionic conductivity of polyelectrolyte films was tested at different humidity levels and temperatures (25 and 40 °C). Moreover, the results of the resistances are shown in Figure S6 (Supporting Information). The ionic conductivity was calculated according to Equation (4).

(4) $$\sigma_{i}=\frac{L}{A\;\times \;R}$$

**Figure 3:**
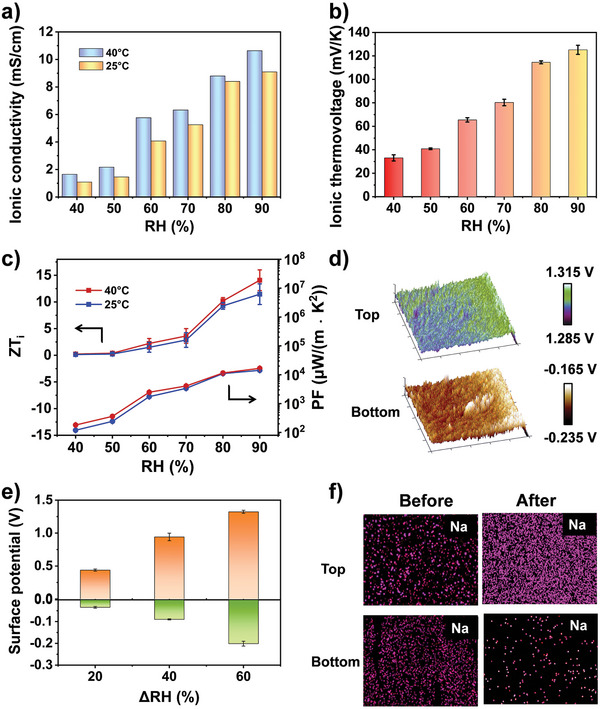
Thermoelectric properties of the P(AMPS‐SSS_0.5_) at different humidity levels. a) Calculated ion conductivities of the P(AMPS‐SSS_0.5_) membrane under different relative humidities at 25 and 40 °C. b) Ionic thermovoltages of the P(AMPS‐SSS_0.5_) membrane under different relative humidities. c) *ZT_i_* and *PF* values of the P(AMPS‐SSS_0.5_) membrane at 25 and 40°C with different humidities. d) Relative surface potentials of the P(AMPS‐SSS_0.5_) membrane (bottom) under 90% RH and 40 °C, and (top) under 40% RH and 25 °C. The area for the KPFM was 1 × 1 µm^2^. e) Relative surface potential of the P(AMPS‐SSS_0.5_) membrane under varying ΔRH. f) Elemental mapping images of Na representing the top (first line) and bottom (second line) before working (left) and after working (right).

Here, *σ*_*i*_ is the ionic conductivity, *L* is the spacing between the two electrodes, *R* is the resistance, and *A* is the area. As shown in **Figure** **3**a, the *σ*_*i*_ of this polyelectrolyte membrane increased with increasing humidity and temperature, from only 1.09 mS cm^−1^ at 40% humidity, and 25 °C, to 10.6 mS cm^−1^ at 90% humidity and 40 °C, showing a higher ionic conductivity than the same type of thermoelectric material (Table S1, Supporting Information). The positive correlation between the ionic conductivity and ionic mobility resulting in a high humidity environment allowed for easier migration of ions in the polyelectrolyte. Table S2, Supporting Information shows the thermal conductivity at different humidity.

The thermovoltage of the P(AMPS‐SSS_0.5_) was measured with the RH and temperature control apparatus (Figure S7, Supporting Information). The positive *S_i_* increased from 32.7 to 126.2 mV K^−1^ under the relative humidity from 40% to 90% (Figure 3b). In thermoelectric materials, the power factor is calculated by Equation (5).

(5) $$PF\;=S_{i}^{2}\;\sigma_{i}$$

As shown in Figure 3c, the *PF* of the P(AMPS‐SSS_0.5_) membrane reached 1.56×10^4^ µW m^−1^ K^−2^ at 90% RH and 40°C, while showed the *ZT_i_* of 14.07 at RH = 90% and 40 °C, and 11.46 at RH = 90% and 25 °C, respectively.

Furthermore, Kelvin probe force microscopy (KPFM) tests with different RH and at different temperatures showed a noticeable surface potential difference between the top and bottom of the P(AMPS‐SSS_0.5_) (Figure 3d). This revealed the adequate ion dissociation and the distribution of heterogeneous charges in the polyelectrolyte membranes after water adsorption under 25 °C, 40% RH and 40 °C, 90% RH. The absolute values of the surface potential difference on both sides of the P(AMPS‐SSS_0.5_) increased with the increasing ΔRH (Figure 3e), signifying a greater dissociation of ions (H^+^ and Na^+^) in the polyelectrolyte membrane. The elemental distribution in MTEG was investigated to confirm the diffusion of the ions during electricity generation. The unequal distribution of Na^+^ in the elemental mapping coincides with the migration of ions and water molecules after working under the hygrothermal synergistic system (Figure 3f). When MTEG absorbed water molecules from the air environment (ΔRH = 60%, Δ*T* = 15 K) for 2 h, the contents of sodium element were about 18.3% and 5.5% at the top and bottom of the membrane, respectively (Table S3, Supporting Information). Comparatively, the Na^+^ contents of the membrane without work were 7.5% and 8.1% at the top and bottom, respectively (Table S4, Supporting Information). The difference in Na^+^ content between the two faces confirmed that Na^+^ migrated from the hot and humid side to the cold and dry side.

### 2.4 Output Performance of the Flexible Power Generating Device

The MTEGs with P(AMPS‐SSS_0.5_) membrane were fabricated to convert thermal water vapor to electricity based on the thermal diffusion effect and dissociation of water molecules. Therefore, the MTEGs perform as the capacitor‐type devices. The output power density was controlled by the output voltage (*V*_output_) and the corresponding current density (*J*_output_) with the same resistance, which is calculated by Equation (6).

(6) $$P_{\mathrm{output}}=V_{\mathrm{output}}\;\times J_{\mathrm{output}}$$

**Figure 4:**
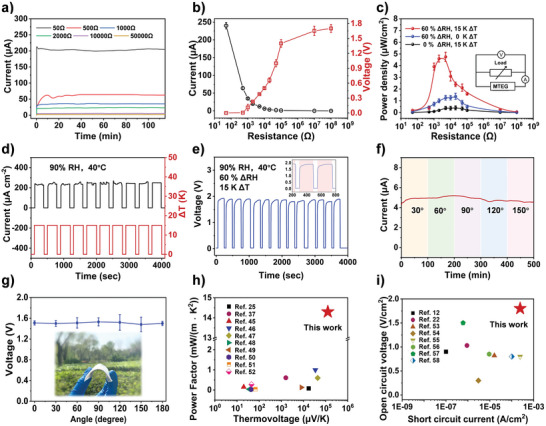
Performances as a capacitor‐type device. a) Real‐time current changes of the as fabricated closed‐circuit with different load resistance at Δ*T* = 15 K and ΔRH = 60%. b) Current–voltage variation of the device under different loads. c) Power density curves of MTEG measured in different environments with a series of loads, and the inset represents the equivalent circuit for the measured condition. Variation of *I*_sc_ d) and *V*_oc_ e) of the device operating cycles in 90% RH and 40 °C by making and removing the gradients of temperature and moisture. The inset in (e) is enlargement from 200 to 800 s. f) Current variation with different banding angles in 500 min. g) Voltage variation at different bending angles. Inset: image of MTEG bending. h) Comparison of the performance of P(AMPS‐SSS_0.5_) with other thermoelectric materials from literature (Refs.[25, 37, 45, 46, 47, 48, 49, 50, 51, 52], Table S6, Supporting Information). i) Comparison of the performance of the MTEG with other MEGs from literature (Refs.[12, 22, 53, 54, 55, 56, 57, 58], Table S7, Supporting Information).

The generation of voltage and current in a closed circuit by varying loads through the circuit is described in **Figure** **4**. The environmental condition is fixed at a temperature difference of 15 K and 60% humidity difference. Remarkably, the current with a constant load resistance of 50, 500, 1000, 2000, 10 000 and 50 000 Ω demonstrated consistent output over 100 min (Figure 4a). The device exhibited an output current of 240 µA at low loads and a *V*_output_ of 1.76 V with a constant load resistance of 10 MΩ (Figure 4b). The results indicate that the closed‐circuit aligned with the regular pattern and works. Figure 4c shows the relationship between the power density and resistance under three different environment conditions. When the device was connected to an optimum resistor of 10 KΩ under 15 K temperature difference and 60% humidity difference, a maximum output volumetric power density of 4.75 ± 0.4 µW cm^−2^ was achieved. The MTEG shows an energy conversion efficiency of 3.0%. Table S5 (Supporting Information) shows the calculated output power density per unit mass, area, and volume. Figure 4d,e show the changes of short‐circuit current (*I*_sc_) and *V*_oc_ when the temperature and humidity differences are artificially given and removed in 4000 s. The reversibility of voltage and current also shows the quasi‐continuous mode of the MTEGs. As soon as the humidity and temperature gradients were removed, the ions diffused freely, resupplying the depleted materials to the electrodes, and the sulphonic acid root re‐attracted the hydrogen ions, thus restoring the battery voltage so that the next discharge cycle can begin. Both current and voltage responded quickly to variations in external conditions, and then the voltage response slows down slightly in the cycles because the humidity difference inside the device is reduced. This quasi‐continuous operation was expected to continue for a more extended period until the electrodes were fully polarized.

Moreover, the flexible MTEG withstood harsh bending deformations and achieved a stable voltage and current retention of 95% at different bending angles from 0 to 180° in 500 min (Figure 4f,g). Such stable performances endow MTEGs with vast applications in various wearable devices, such as masks and bracelets. The thermoelectric and moist‐electric performances of the device were compared with those in the literature. The ionic thermovoltage and power factor were measured, and they showed large increments (Figure 4h).^[^ ^24^, ^37^, ^45^, ^46^, ^47^, ^48^, ^49^, ^50^, ^51^, ^52^ ^]^ Similarly, the MTEG was operated in a hygrothermal synergistic system, and its areal *V*_oc_ and *I*_sc_ were compared with related literatures (Figure 4i).^[^ ^12^, ^22^, ^53^, ^54^, ^55^, ^56^, ^57^, ^58^ ^]^ More details are shown in Tables S6 and S7 (Supporting Information). Since sufficient voltage and power levels were achieved under the hygrothermal synergistic system, the high efficiency of the MTEG under the combined environments was much higher than the values reported in previous works on MEGs and TEGs (Figure S8, Supporting Information).

### 2.5 Integration of Power Generating Device

**Figure 5:**
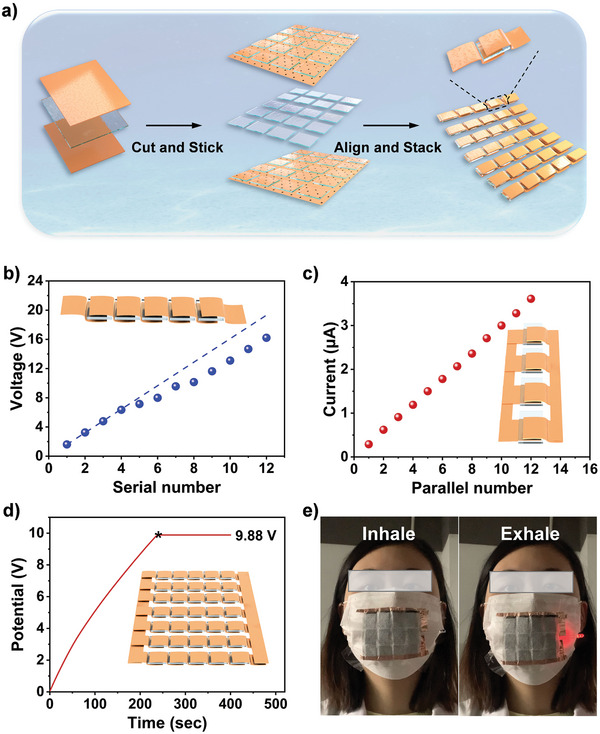
Device integration methods and performance. a) Process of integrating the scalable electrical generators. b) Variation of the *V*_oc_s of series integrated devices, inset is the device diagram. c) Variation of the *I*_scS_ of parallel integrated devices, inset is the device diagram. d) *V*_oc_‐time curve for an integrated device with an effective area of 7.5 cm^2^. e) Images of the integrated MTEGs lighting up a small bulb using the hot moisture from human breath.

**Figure 6:**
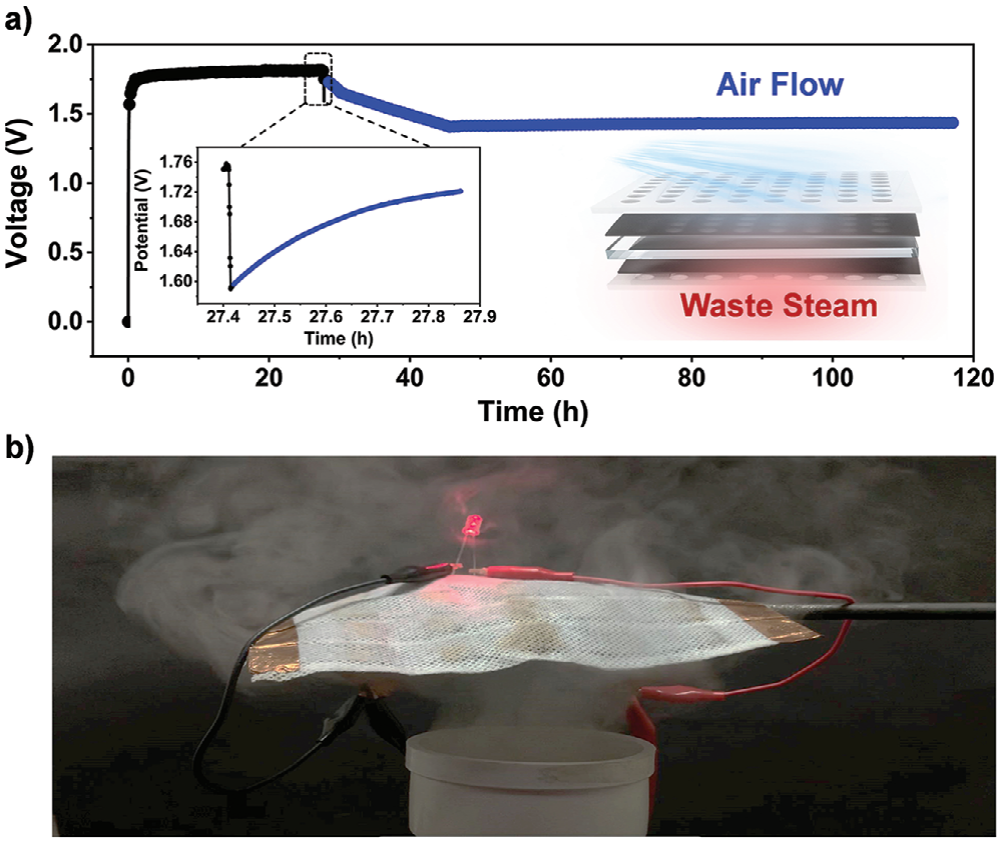
Device performance in practical applications. a) Voltage output of the MTEG is sustained for 118 h under 60% ΔRH and 15 K temperature difference (Environment temperature at 70 °C), the top of device is the given gas flow after 27 h. b) Image of the device lighting up a LED bulb by a waste steam outlet.

The scalable integration of electric generators is critical for realizing high‐performance power outputs in ambient environments. Flexible and sizable integrated devices have wide applications in wearable devices, and we integrated the MTEG units on a large scale. After the whole carbon cloth sheets and large areas of the polyelectrolyte membrane were prepared, they were cut to the required sizes, arranged, and stacked in order of pole, electrolyte and pole to form the integrated device (**Figure** **5**a). The output of the integrated device increased linearly with the number of serial units, confirming the scaling performance of the device. The integrated device identical in 12 units offered a voltage of up to 15.97 V (Figure 5b). As the number of integrations increased, the voltage did not completely increase linearly, presumably because of the increase in internal resistance. Additionally, the current was steadily scaled up by rational parallel connections and remained stable in 100 min (Figure 5c; Figure S9, Supporting Information). The more complex series‐parallel integrated device also achieved an actual voltage of 9.88 V. The sum of the area of electrodes was 7.5 cm^2^ (Figure 5d). The scalable integration method is shown in the inset of each graph. The integrated device with an effective area of 3 × 3 cm^2^ was able to light up a red LED bulb (>2.3 V, 800 µA) by the hot moisture produced from boiling water (Figure S10, Supporting Information) without any other auxiliaries. Meanwhile, the integrated MTEGs show an energy conversion efficiency of 0.67% (effective area: 9 cm^2^). The integration product was folded, bent, and attached to the outside of a mask (Figure S11, Supporting Information). The decorated mask lit up a red LED bulb with the breath of humans at a fast response time (Figure 5e; Video S1, Supporting Information). The heat from hot moisture encouraged water evaporation and prevents moisture saturation, thereby, the MTEG can continuously work for 27 h with a *V*_oc_ of ≈1.8 V. The voltage decreased slowly due to the reduced humidity difference. However, it was capable to go back and became stable at about 1.41 V until 118 h as the given air flow (**Figure** **6**a). The device's capabilities in practical applications are demonstrated in Figure 6b, where it can convert waste steam energy to light a light bulb. These results suggest that the MTEGs successfully charge the electrical appliances in high temperature and high humidity environments, broadening the scenes in that TEGs and MEGs can be operated. In addition, MTEGs can be used as power supplies in industrial processes with waste steam energy, and wearable devices with energy exhaled by humans.

## 3 Conclusion

In summary, we have designed a “two‐in‐one” MTEG using flexible polyelectrolyte membrane P(AMPS‐SSS_0.5_). The MTEG generated electricity at a voltage output of 1.81 V in a synergistic hygrothermal environment (ΔRH = 60%, Δ*T* = 15 K), based on the spontaneous adsorption of low‐grade heat moisture and induced diffusion of H^+^ and Na^+^. It also exhibited a high *S_i_* of 126.2 mV K^−1^, high *σ*_*i*_ of 10.6 mS cm^−1^, low *κ* of 0.3709 W m^−1^ K^−1^, and a *ZT_i_* of 14.07 ± 1.93 under a relative humidity of 90% and 40°C. The power density was 4.75 ± 0.4 µW cm^−3^. The MTEG exhibits the highest thermovoltage and power density than polyelectrolyte membrane‐based TEG or MEG reported previously. Additionally, the integrated MTEGs can supply power for medium and large equipment by conversion the energy of industrial waste steam. The unique MTEG presented here offer a versatile option for the promotion of green and sustainable power generation.

## 4 Experimental Section

### Preparation of the Polyelectrolyte Membranes

Sodium styrene sulfonate (SSS) and 2‐acrylamide‐2‐methyl propane sulfonic acid (AMPS) (molar ratio of 2:1) were dissolved in water. The crosslinking agent N, *N*’‐methylene bisacrylamide (MBAA) with photoinitiator *α*‐Ketoglutaric acid in a molar ratio of 1% monomer was added in the solution with an overall water content of 70%. Then pour it into a mould of polytetrafluoroethylene. The polymerization was carried out under UV light (wavelength 365 nm) for 30 min, followed by drying in an oven at 80 °C for 15 min. The P(AMPS‐SSS_0.5_) membrane was cut into a 2 × 2 cm^2^ size for subsequent use P(AMPS‐SSS_1_) and P(AMPS‐SSS_2_) membranes were also prepared as described above.

### Preparation and Integration of MTEG (Moist‐Thermoelectric Generator)

Water‐permeable adhesive tape (thickness of 200 microns) was used as a substrate for manufacturing flexible P(AMPS‐SSS_x_). Ventilation holes distributed in a matrix arrangement were fabricated on the tape. The hydrophilic carbon fabric (CeTech W1S1011) glued to adhesive tape as the electrode. The P(AMPS‐SSS_x_) was sandwiched between two decorated tapes, and the conductive position was led with copper glue to facilitate the next testing step. The MTEG units were further connected in series and parallel to achieve the desired integrated MTEG.

### Characterizations of P(AMPS‐SSS_x_) Polyelectrolyte Membranes

The morphology and corresponding chemical composition analysis were conducted on a scanning electron microscope (SEM) (Hitachi Regulus 8230, Japan) equipped with an energy dispersive spectrometer (EDS) (OXFORD Ultim Extreme EDS, UK). Fourier transform infrared (FTIR) spectra were tested on a Varian CP3800 spectrometer instrument with a wavenumber range of 4000–600 cm^−1^. Kelvin probe force microscopy (KPFM) was conducted on a Bruker Multimode8 machine. Specific heat (*C*_P_) was characterized using Different Scanning Calorimetry (DSC 4000) at 223–323 K.

### Thermoelectric Properties Characterization

The ionic conductivity (*σ*_*i*_) of the P(AMPS‐SSS_x_) membranes were determined by AC‐impedance spectroscopy with an Autolab potentiostat galvanostat (Metrohm). The voltage amplitude was 10 mV, and the frequency was scanned from 100 to 0.1 Hz. The P(AMPS‐SSS_0.5_) membrane was sandwiched with an area of 3.04 cm^2^ between two stainless steel plates. The space between two electrodes was ≈600 µm, measured with a spiral micrometer.

The thermal conductivities of the P(AMPS‐SSS_x_) membranes were determined using a TC_i_ thermal conductivity analyzer (C‐therm). The membrane was prepared by drop‐casting with a thickness of 600 µm. The principle of the thermal conductivity analyzer is the transient heating method, and the thermal conductivity is obtained by monitoring the temperature change with time. The transient heater located at the center, and the temperature change at the surrounding is measured. Thermoelectric properties were measured at room temperature and relative humidity from 40% to 90%. The thermal conductivities were measured three times at the same relative humidity.

### Electric Measurement

All the voltage and current signals were recorded in real‐time using a Keithley 2612 multimeter controlled by a LabView‐based data acquisition system. All the samples were short‐circuited before testing to avoid any inference from the static electricity. The bias voltage for testing short‐circuits current was ≈1 µV, and the bias current for testing the open‐circuit voltage was set to be ≈1 × 10^−11^ A. The energy stored in the capacitor was measured and calculated by discharging the capacitor with a galvanostatic technique using an electrochemical workstation (CHI 760C, China). Based on the definition of the ionic thermovoltage, the thermovoltage is calculated in terms of the *V*_oc_ and the temperature difference. In this study, the ion thermovoltage, the output power density and the bending voltage of the MTEGs were measured three times. The error bars are the standard deviations of these data.

### KPFM Test

The one P(AMPS‐SSS_0.5_) membrane (600 µm in thickness) was stored in an incubator with a constant RH at 90% and a constant temperature of 40 °C for 2 d to ensure the homogeneous distribution of water molecules in the membrane. The other one was stored in an incubator with a constant RH at 30% and a constant temperature at 25 °C simultaneously. The potential of two pieces of the membranes was tested on a KPFM machine. One side of the membrane has been simulated the condition of the top side of the MTEG during working (which is relatively dry and cold) and the other side was the condition of the bottom side (which is relatively hot and wet). Therefore, the difference of the surface potential is that under Δ*T* = 15 K and ΔRH = 60%. The potential change of the top and the bottom sides was measured by KPFM with different incubators owing different environments, respectively.

## Conflict of Interest

The authors declare no conflict of interest.

## Author Contributions

M.Y. and F.Y. conceived the concept. Y.H. and F.Y. supervised the project. M.Y. and Y. H. performed the experiments and analyzed the data. S.Z., Z.L., and W.L. participated in optimizing the figures. M.Y., Y.H., and F.Y. wrote the manuscript. All authors discussed the results and commented on the manuscript.

## Supporting information

Supporting Information

Supplemental Video S1

## Data Availability

Research data are not shared.
