# Supplementary material for: Integrated Moist‐Thermoelectric Generator for Efficient Waste Steam Energy Utilization
Source: Adv Sci (Weinh). 2023 May 28;10(22):2206071. doi: 10.1002/advs.202206071 (PMC10401182; doi:10.1002/advs.202206071)
Supplement: Supplementary file 1 — Supporting Information [file ADVS-10-2206071-s002.pdf]

ADVANCED SCIENCE
Open Access

Supporting Information

for *Adv. Sci.*, DOI 10.1002/advs.202206071

Integrated Moist-Thermoelectric Generator for Efficient Waste Steam Energy Utilization

Mingchen Yang, Yin Hu, Sijie Zheng, Ziyang Liu, Weizheng Li and Feng Yan**

Supporting Information

Integrated Moist-thermoelectric Generator for Efficient Waste Steam Energy Utilization

Mingchen Yang, Yin Hu, Sijie Zheng, Ziyang Liu, Weizheng Li and Feng Yan**

Jiangsu Engineering Laboratory of Novel Functional Polymeric Materials,

Jiangsu Key Laboratory of Advanced Negative Carbon Technologies College of Chemistry,

Suzhou Key Laboratory of Soft Material and New Energy, College of Chemistry, Chemical Engineering and Materials Science, Soochow University, Suzhou 215123, China.

E-mail: yinhu045@suda.edu.cn and fyan@suda.edu.cn

This file includes:

Materials

Supplementary Text

Figures S1 to S11

Tables S1 to S7

Other Supplementary Materials for this manuscript include the following:

Videos S1

Materials

Sodium styrene sulfonate (SSS), 2-acrylamide-2-methyl propane sulfonic acid (AMPS), N, N'-methylene bisacrylamide (MBAA) and α-Ketoglutaric acid were purchased from Sigma-Aldrich. Water-permeable adhesive tape was purchased from Hons Medical. The hydrophilic carbon fabric W1S1011 was pruchased from Ce-tech Co., Ltd. Deionized water was utilized throughout all the experiments.

Supplementary Text

Supporting notes for calculation of the energy conversion efficiency

For the MTEG unit (1 cm^{-2}), the mass of the polyelectrolyte membrane increased from 0.079 to 0.083 g after one hour of operation under the synergistic system of gradient temperature and humidity (100% RH for the bottom and 40% RH for the top, and 25 °C for the top and 40 °C for the bottom). The change in the mass was due to the adsorption of water during the operation. In the process of water adsorption induced electricity generation of MTEG, the variation of the chemical potential energy of water molecules, corresponding to the transformation from gaseous water in the air to adsorb water in MTEG, could be reasonably considered as the main energy source. In response to water molecules adsorption of MTEG, oppositely charged ions (i.e., H^+ and Na^+) will be dissociated. They could diffuse in opposite directions based on the concentration difference effect, thereupon generating electric output on an external circuit. Therefore, the input energy finally is converted into the electric energy of MTEG. For energy conversion efficiency in the electricity generation process, the chemical potential of gaseous water and adsorbed water is μ_g and μ_a, respectively. The MTEG enables to spontaneously adsorb gaseous water in the air, and the water adsorption is assumed to be an isothermal and isobaric process. From thermodynamic law, the normal chemical potential μ_i is calculated as[S1]

$$\mu_i = \left(\frac{\partial G}{\partial n_i}\right)_{T,P} \qquad (S1)$$

$$\mu_i = \mu_i^{\theta} + RTIna_i \quad (S2)$$

where G, n_i, T, P, R, μ_i^{θ} and a_i represents Gibbs free energy, the number of moles, temperature, atmospheric pressure, ideal gas constant, standard chemical potential and activity, respectively. In the process of water adsorption of MTEG, the water molecules will spontaneously change from a free gaseous state to an adsorbed state. The Gibbs free energy variation can be considered as:

$$\Delta G = \mu_a - \mu_g < 0 \quad (S3)$$

which reflects the reduction of the chemical potential energy of water molecules. Because there is no additional energy input in this MTEG system (single humid case), the chemical potential variation of water can be considered the sole energy input for electricity generation. Thus, the maximal energy input could be estimated as:[S2]

$$\Delta G = \mu_a - \mu_g \approx RTln\frac{c_0}{c_0 - \Delta c} \quad (S4)$$

where c_0 and Δc represents the concentration of water in atmosphere and the concentration variation of water, respectively. The concentration of water in atmosphere and the concentration variation of water could be estimated by relative humidities of two sides. As a result, we could appropriately calculate the maximal energy input of about 0.305 J at 313 K arising from variation in chemical potential of water.

Meanwhile, the temperature gradient of two sides of the polyelectrolyte membrane induces the Na^+ immigration due to the Soret effect. In the process of thermal migration, the internal energy of the water is reduced, supplying the ions to diffuse and migrate and thus generate further electrical energy. The temperature change of the water (condensed matter), in MTEG, is assumed to be an isobaric and isotropic process.

From thermodynamic law, the change of internal energy of water is calculated as:

$$\Delta U = \Delta Q + W \quad (S5)$$

$$\Delta Q \approx \Delta H = nC_{P.m}(T_2 - T_1) \tag{S6}$$

$$W \approx 0 \tag{S7}$$

where ΔQ and W represent the heat absorbed by the object and the work done on the object, respectively. The calculated maximal energy input under the isobaric and isotropic cooling process was about 0.258 J. In summary, the total energy input during the entire operation is about 0.563 J. In the synergistic system of gradient temperature and humidity, the P_{max} is 4.75 μW cm^{-2}.

The energy of MTEG has been measured by connecting an optimally external resistor. The generated electric power can be calculated as:

$$W = \int U(t)I(t)d(t) \tag{S8}$$

where U, I, and t are the generated voltage, current, and time of producing electricity, respectively. As a result, the calculated electricity energy is about 0.0171 J in one hour (100% RH for the bottom and 40% RH for the top, and 25 °C for the top and 40 °C for the bottom). Accordingly, the energy conversion efficiency ($W/\Delta G+\Delta Q$) is estimated to be about 3% for one MTEG unit.

For the integrated MTEGs which are used in the utilization of the waste steam (60% ΔRH and 15 K temperature difference, environment temperature at 70 °C), the output power of the device is about 28.9 μW (effective area = 9 cm^{-2}). After one hour of operation, the Δm of polyelectrolyte is 0.07434 g. Therefore, the input energy and the output energy are about 15.4356 and 0.10404 J, respectively. Accordingly, the energy conversion efficiency ($W/\Delta G+\Delta Q$) is estimated to be about 0.67% for the integrated MTEGs (effective area = 9 cm^{-2}) under 60% ΔRH and 15 K temperature difference. The decrease in conversion efficiency of integrated MTEGs is attributed to the increase in internal resistance due to the increased number of integrations.

Supporting notes for the Nyquist plots of the polyelectrolyte

The ionic resistances are the intersection of the line with the x-axis.[S3-5] The Nyquist plots of the polyelectrolyte with different RH under different temperatures are shown in Figure S6. We measured the *L* (600 μm), R (shown in **Table S1**), and A (3.04 cm^2) to calculate the ionic conductivity.

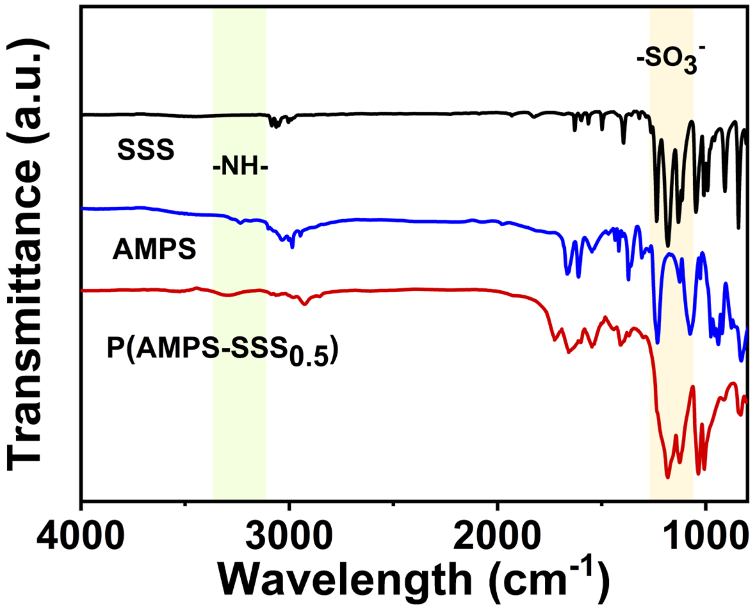

Figure S1. ATR-FTIR spectroscopy of AMPS, SSS, and P(AMPS-$SSS_{0.5}$) membrane.

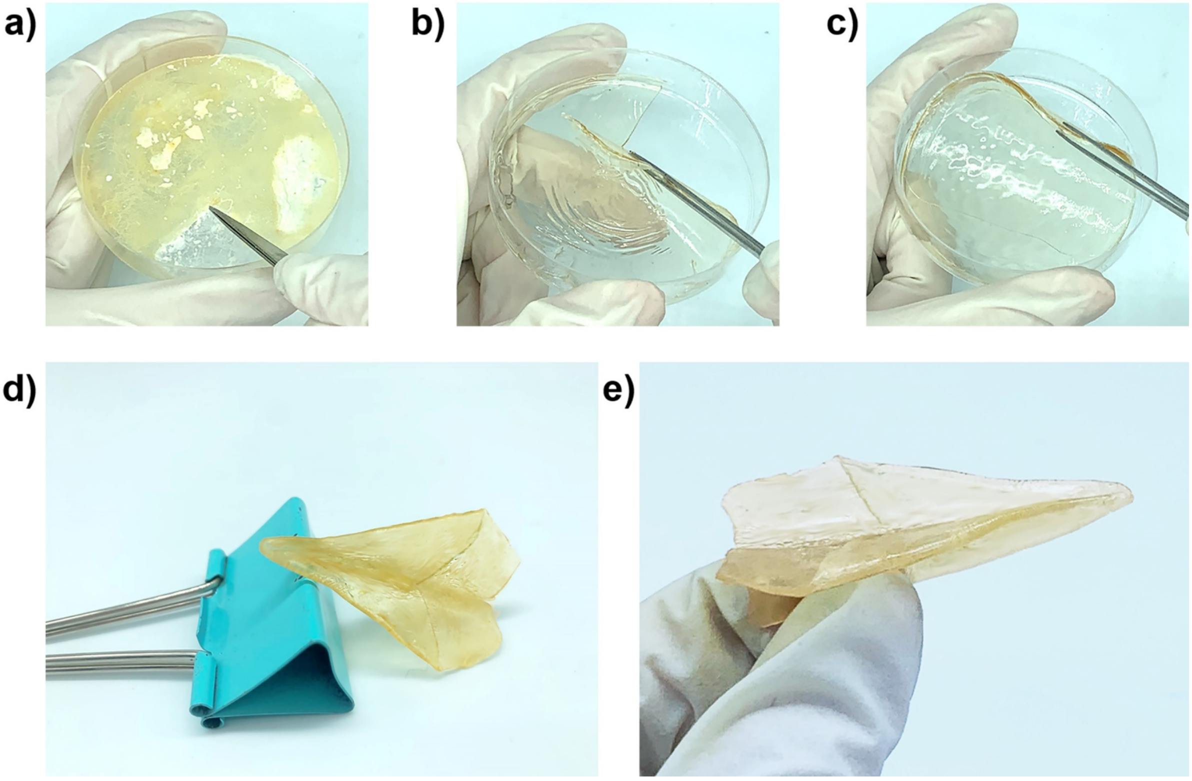

Figure S2. Images of P(AMPS-SSS_x) membranes with different molar ratios of SSS. (a) P(AMPS-SSS_2), (b) P(AMPS-SSS_1) and (c) P(AMPS-$SSS_{0.5}$). P(AMPS-$SSS_{0.5}$) membrane maintains good mechanical properties which can be peeled off from the PET mold and folded as a model plane (d, e).

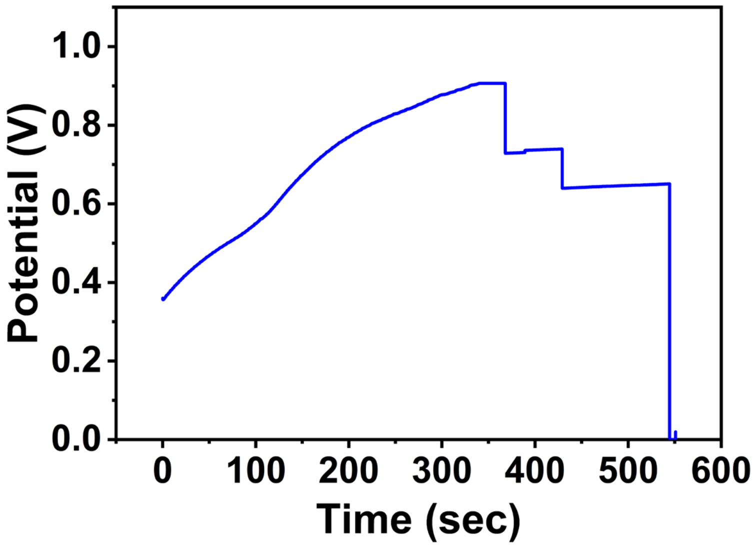

Figure S3. Open-circuit potential curve of the MTEG mounted by the P(AMPS-SSS_1) membrane.

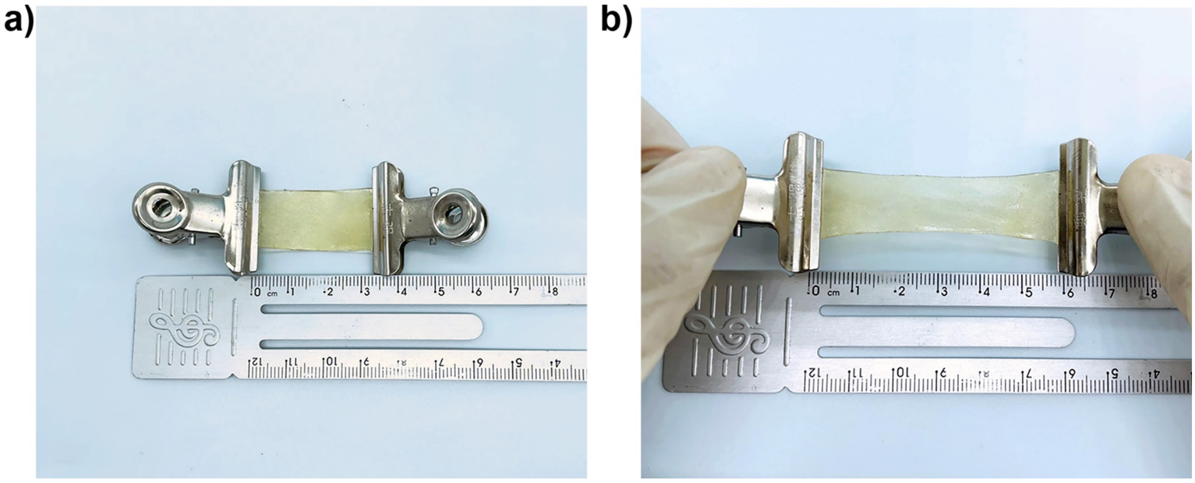

Figure S4. Images of P(AMPS-$SSS_{0.5}$) membrane with (a) normal and (b) twice stretched conditions. The P(AMPS-$SSS_{0.5}$) membrane shows high mechanical strength and flexibility.

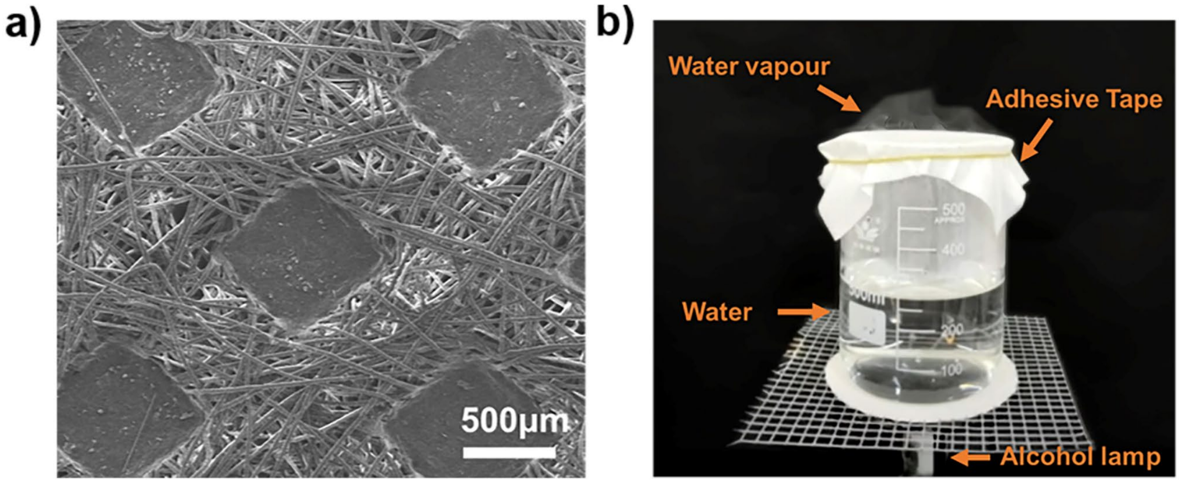

Figure S5. (a) SEM image of the holes of hydrophilic adhesive tape. (b) Image of the water-permeable and breathable effect of hydrophilic adhesive tape to demonstrate the water permeability.

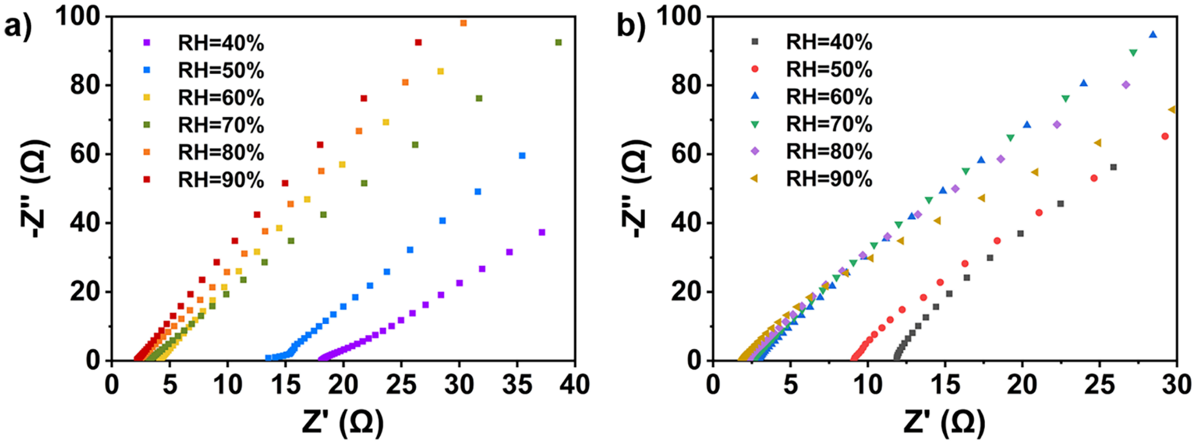

Figure S6. Nyquist plots of P(AMPS-$SSS_{0.5}$) membrane at different relative humidity levels at (a) 25 °C and (b) 40 °C.

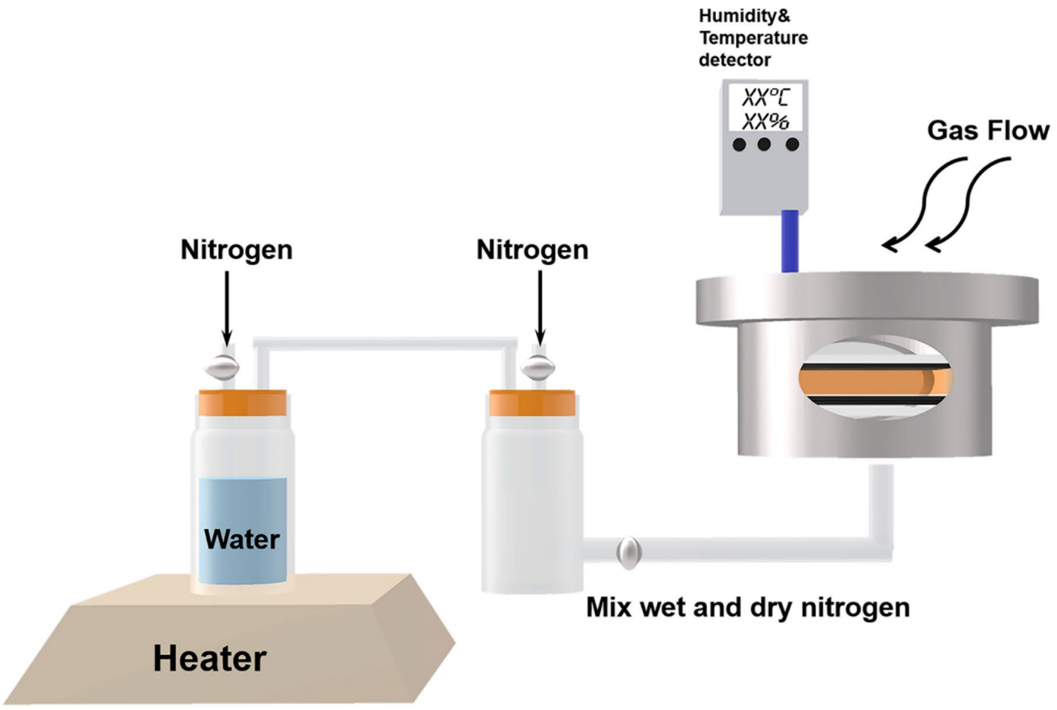

Figure S7. Apparatus for the temperature and RH control. Nitrogen passes through the water at different temperatures and brings out water vapor at different temperatures, while ΔRH is controlled by mixing dry and wet nitrogen gas with different ratios and monitored by a commercial humidity sensor. The gas flow controls the temperature and humidity above.

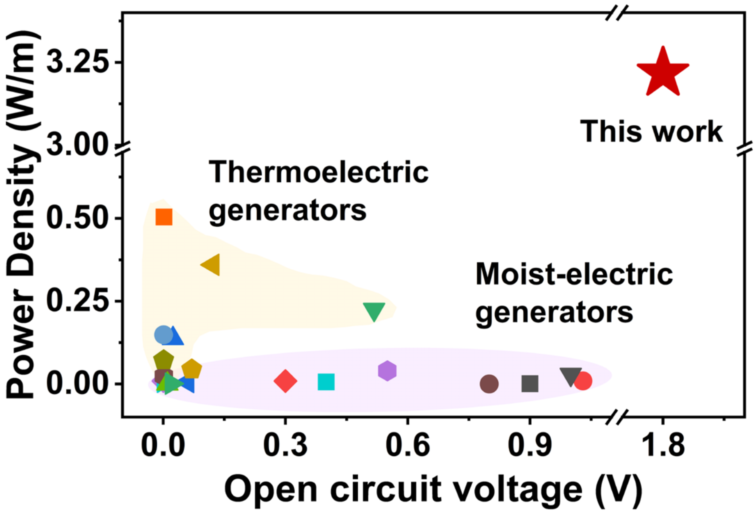

Figure S8. Systematic performance comparison of the reported thermoelectric generators and moist-electric generators.

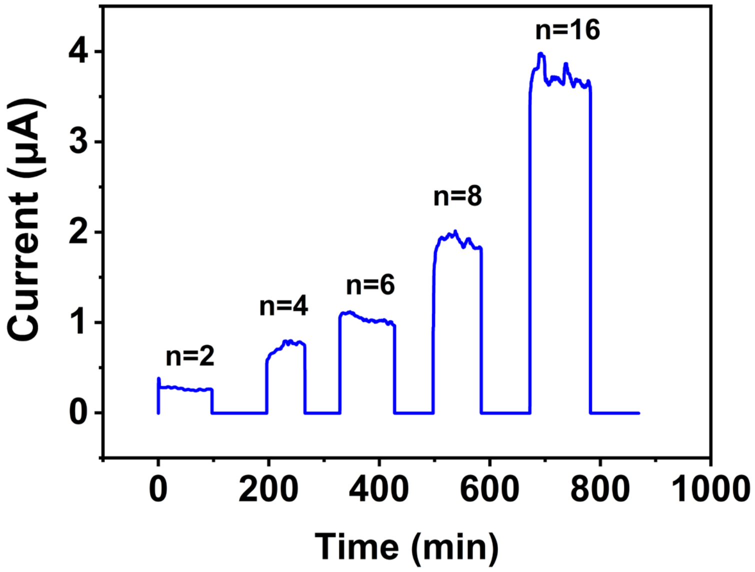

Figure S9. Variation of I_{sc} of different parallel numbers of integrated devices in 100 min. The n represents the parallel number. The environment condition is 15 K of ΔT and 60% ΔRH.

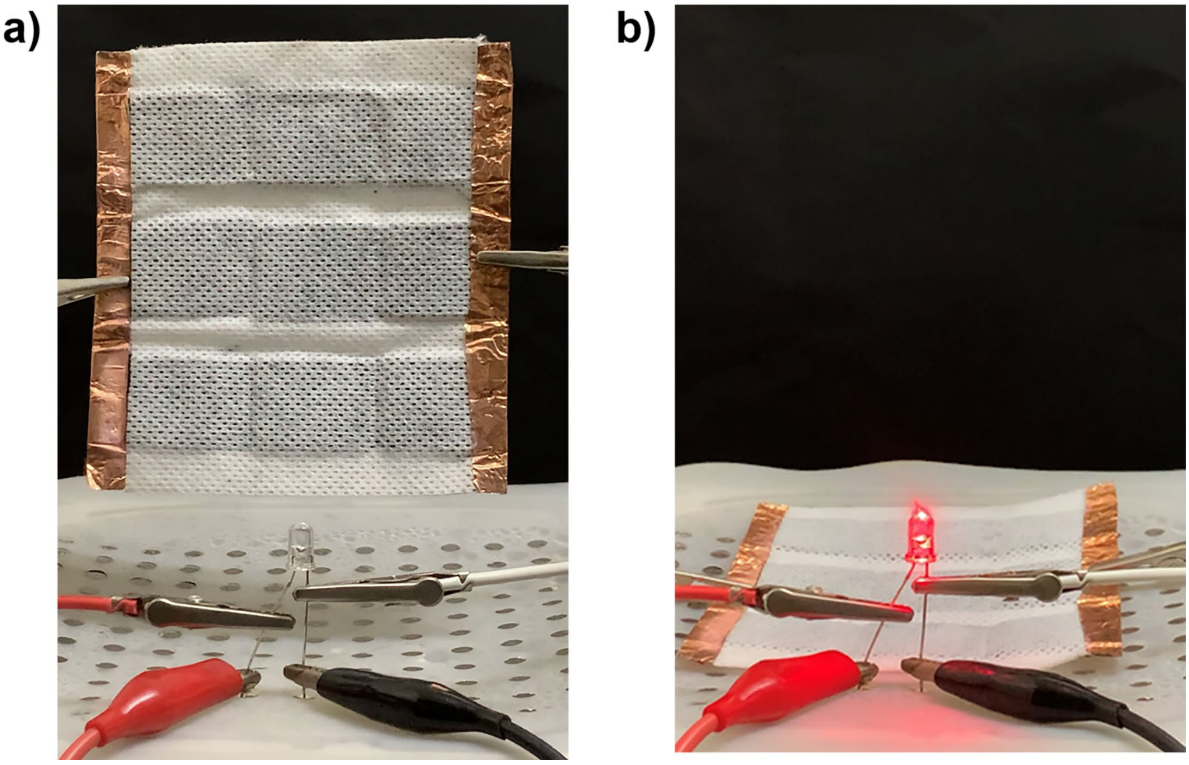

Figure S10. (a) Images of the device of integrated MTEGs and (b) the device lightening the red LED bulb by hot moisture from the boiling water.

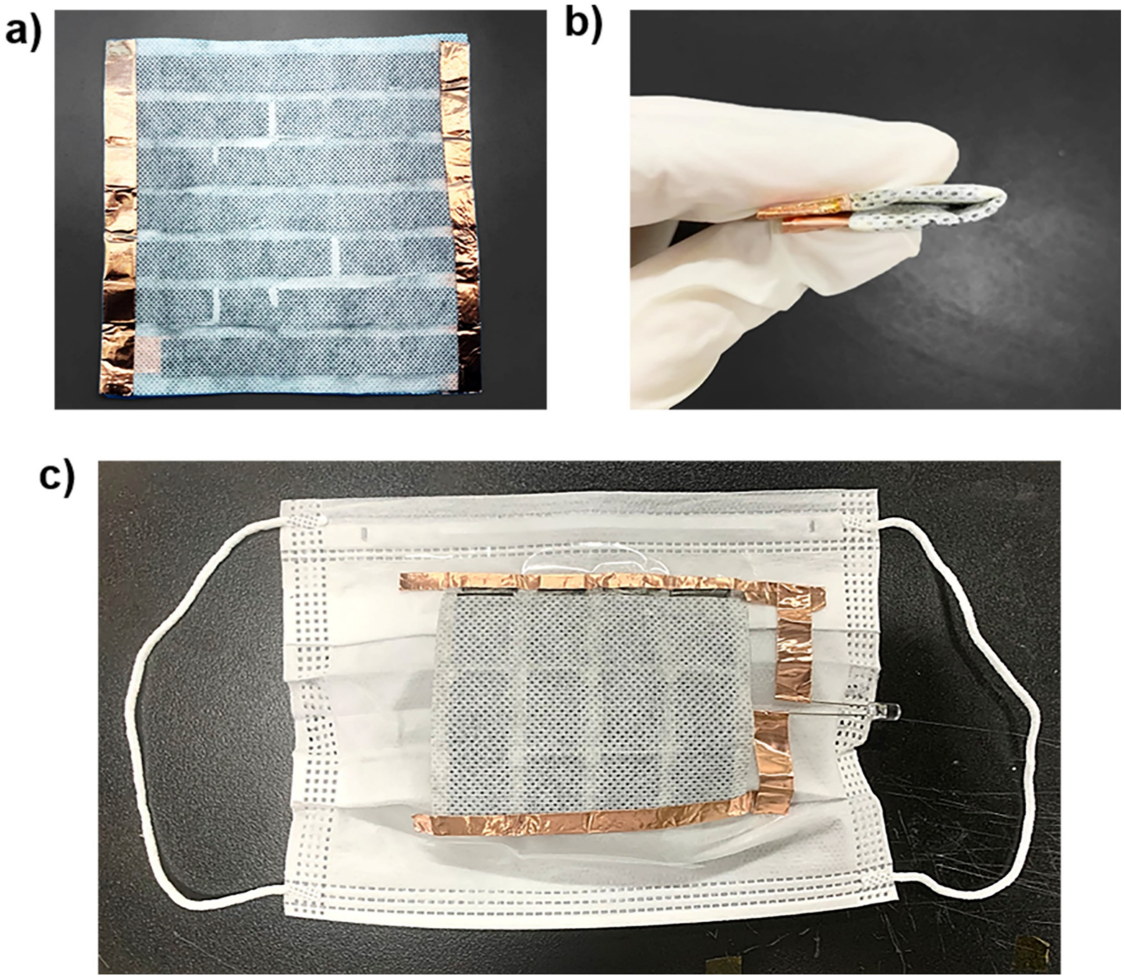

Figure S11. Images of integrated MTEGs in the (a) normal state, (b) folded state and (c) attached to the mask.

Table S1. Calculated ionic conductivities of the P(AMPS-$SSS_{0.5}$) with different humidities at 25 °C and 40 °C.

RH (%)	40%	50%	60%	70%	80%	90%
Resistance (Ω) 25 °C	18.072	13.512	4.845	3.754	2.348	2.171
Resistance (Ω) 40 °C	11.902	9.076	3.422	3.121	2.243	1.856
Ionic conductivity (mS cm^{-1}) 25 °C	1.093	1.462	4.074	5.257	8.405	9.096
Ionic conductivity (mS cm^{-1}) 40 °C	1.659	2.175	5.769	6.326	8.803	10.638

Table S2. Thermal conductivities of the P(AMPS-$SSS_{0.5}$) at relative humidities.

Relative Humidity (%)	40	50	60	70	80	90
κ [W/(m·K)]	0.24483	0.29823	0.34793	0.35287	0.35483	0.37087
Standard deviation	0.00389	0.000833	0.00358	0.00869	0.000252	0.00626

Table S3. Elemental content analysis results for the two sides of the P(AMPS-$SSS_{0.5}$) after work.

	Element	Carton (C)	Oxygen (O)	Nitrogen (N)	Sulfur (S)	Sodium (Na)
Top	At%	45.5	21	8.2	7	18.3
Bottom	At%	51.9	24.5	10.5	7.7	5.5

Table S4. Elemental content analysis results for the two sides of the P(AMPS-$SSS_{0.5}$) without work.

	Element	Carton (C)	Oxygen (O)	Nitrogen (N)	Sulfur (S)	Sodium (Na)
Top	At%	52.2	23.4	10	6.9	7.5
Bottom	At%	49.1	25.8	9.7	7.3	8.1

Table S5. A series of related performance parameters of the MTEG in a closed circuit. (ΔRH=60%, ΔT=15 K)

	Output power density	Output current	Output voltage
Gravimetric	62.81 μW g^{-1}	198.68 μA g^{-1}	22.52 V g^{-1}
Areal	4.75 μW cm^{-2}	240.02 μA cm^{-2}	1.70 V cm^{-2}
Volumetric	95.00 μW cm^{-3}	4800.4 μA cm^{-3}	34.10 V cm^{-3}

Table S6. The performance comparison between the prepared MTEG and the representative TEGs that have been reported in recent years.

	Thermovoltage ($\mu V\ K^{-1}$)	Power factor ($mW/(m\ K^2)$)	Type
This work	126000	14.3	Polyanionic membrane
Science, 2020, 368, 1091–1098	17400	0.06	Ionic gelation
Joule, 2021, 5, 2211–2222	1600	0.61	Redox thermoelectric hydrogel
Adv. Funct. Mater.,2020, 30, 2004699.	34500	0.9998	[EMIM:DCA] with WPU
Nat. Commun., 2022, 13, 221.	20	0.15	p-type i-TE PhNP composites
Adv. Energy Mater., 2022, 12, 1–8	22	0.019	PPy/SWCNT composite films
Chem. Mater., 2019, 31, 3519–3526.	35	0.04	PEDOT:PSS/Ionic Liquid Composites
Energy Environ. Sci., 2020, 13, 2915–2923	8100	0.13	PANI:PAAMPSA:PA
Nat. Commun., 2020, 11,1424	44	0.03	PEDOT:PSS-WPU-IL
Nat. Commun.,2020, 11, 572	68	0.04	Woven thermoelectric fiber
CCS Chem., 2021, 3, 2415–2427	45	0.28	PEDOT:PSS and Bi_2Te_3 with DMSO vapor

Table S7. The performance comparison between the prepared MTEG and the representative MEGs that have been reported in recent years.

	Open circuit voltage (V cm^{-2})	Short circuit current (A cm^{-2})	Environment	Type
This work	1.81	2.4E-4	ΔRH=60%, ΔT =15 K	Polyanionic membrane
Energy Environ. Sci., 2018, 11, 1730–1735.	0.9	1E-7	ΔRH=70% ,R.T.	Graphene oxide (GO) functionalized paper
Nat. Nanotechnol., 2021, 16, 811–819	1.03	9E-7	85% RH., R.T.	Polyelectrolyte films
Nano Energy, 2020, 68, 104364	0.82	1.6E-5	RH=82%, R.T.	A graphitic carbon layer
Nano Energy, 2018, 46, 297–304	0.3	3E-6	ΔRH=75% ,R.T.	Gradient doped polypyrrole nanowire
Adv. Mater.,2022, 34, 2200693	0.8	2.4E-4	RH=60% ,R.T.	PVA-PA-glycerol
Nano Energy, 2022, 94, 106942	0.85	9.28E-6	RH=75% ,R.T.	GO/PVA,
Nano Energy, 2022, 94, 106917	1.5	6E-7	RH = 60%,R.T.	Silicon nanowires
Energy Environ. Sci., 2019, 12, 972–978	0.8	1E-4	ΔRH=85% ,R.T.	PSSA film

Supporting Video 1.

The integrated MTEG attached to the outside of the mask uses the hot moisture energy of exhaled breath to light up the LED bulb.

References

[S1] M. K. Brachman, *J. Chem. Phys.* **1954**, *22*, 1152.

[S2] S. I. Lukyanov, Z. S. Zidib, S. V. Shevkunovc, *J. Mol. Struct. THEOCHEM* **2003**, *623*, 221.

[S3] Y. Fang, H. Cheng, H. He, S. Wang, J. Li, S. Yue, L. Zhang, Z. Du, J. Ouyang, *Adv. Funct. Mater.* **2020**, *30*, 2004699.

[S4] X. He, H. Cheng, S. Yue, J. Ouyang, *J. Mater. Chem. A* **2020**, *8*, 10813.

[S5] H. Gao, B. Guo, J. Song, K. Park, J. B. Goodenough, *Adv. Energy Mater*. **2015**, *5*, 1402235.
